# Health Tract, No. 222

**Published:** 1864-10

**Authors:** 


### HEALTH TRACT, No. 222.

### HORSE RATIONS.

The horses of the Third Avenue Railroad Company of New York City travel twenty miles a day over almost a dead level of cobble stone pavement, and being checked and started every five minutes or oftner, it becomes a laborious service; to keep them in good condition, and maintain their efficiency, has required a great deal of reflection, judgment and observation. The regular daily rations of each animal are: Sound corn meal, 17 lbs.; good clean hay, mostly timothy, 13 lbs. The hay is chaffed in a cutting machine driven by horse-power, and wet and mixed with the meal, and fed in regular messes at regular times as nearly as the service will permit.—Of course there is no waste of food and no using good hay for bedding. That is made of rye straw, which may average about three pounds a day per horse. The feed boxes are carefully examined after feed, to see that all has been eaten, and to prevent any accumulation of sour or damaged food, and if a horse is "off his feed," to remove him at once to the hospital. The stall and feed boxes are kept constantly as clean as brushes, brooms, and Croton water can make them. The company keep seven hundred horses.

A family carriage horse, a "hack" for the neighbors also, five years old, keeps lively, healthy and fat by being fed thrice a day as follows:

Morning a bushel basket of cut oat straw, four quarts of "shorts" mixed thoroughly after being moistened with water.

Noon, same amount of straw and three quarts of shorts, clear.

Night, a bushel basket of hay and straw, half and half, cut, mixed with four quarts of shorts. He had in addition, the pairings of potatoes and apples and cabbage leaves from the family kitchen. This is believed to be a saving of one half, compared with the ordinary method of feeding horses. The London Omnibus Company feeds six thousand horses daily; they found that horses fed with sixteen pounds of bruised oats, seven and a half pounds of cut hay, and two and a half pounds of cut straw, looked as well, and did as much work on their nineteen pounds of food daily, as on twenty-six pounds a day, if the oats were *not* bruised and the hay and straw were not cut; a difference of three hundred dollars a day. It is perhaps most economical in this country to feed work horses with fine ground oat and corn meal, two-thirds of the former and one-third of the latter well mixed, the former contains the muscle forming principle, the power to labor, the latter the fat, the carbon, for warmth.

But what has "Horse Flesh" to do with human health? "Much every way." "On horseback" is one of the most healthful, graceful and manly of all forms of exercise. It is exhilarating to mind and body; there is not a muscle in the human system which is not brought more or less into requisition, and the great danger is obviated, especially to invalids, of exhaustive exercise, and of being over heated and thus made liable to fresh colds; besides, it involves the breathing of a pure atmosphere all the time; itself an all important element to health seekers; and the difference between riding or driving a sleek charger, and a bag of bones, is as wide as the poles asunder. A man on a poor horse feels as mean as if he had on a dirty shirt, or a hole in his stocking, or as if he hadn't a dollar in his pocket. There is more health to the cabined, cribbed and confined daughters of citizens and farmers in one hour's daily horse-back riding, about sun rise, for nine months in the year, with gentlemen of refinement, courteous and joyous nature, than in all the drugs and "pathies" in the universe. Besides, we owe it to the noblest of all animals to understand how to take the best care of him.

